# Comparison of Interferon‐Based and Interferon‐Free Treatments on the Prognosis of Hepatocellular Carcinoma After Hepatitis C Virus‐Sustained Virological Response: A Multicenter Study

**DOI:** 10.1002/cam4.71963

**Published:** 2026-06-11

**Authors:** Shinji Itoh, Norifumi Iseda, Tomoharu Yoshizumi, Takao Ide, Hirokazu Noshiro, Hisamune Sakai, Toru Hisaka, Masao Nakashima, Hiroaki Nagano, Hirohisa Okabe, Hiromitsu Hayashi, Atsushi Miyoshi, Kenji Kitahara, Yuko Takami, Atsushi Nanashima, Tamotsu Kuroki, Yuichi Endo, Kohji Okamoto, Masatoshi Kajiwara, Masahiko Sakoda, Toru Beppu, Mitsuhisa Takatsuki, Susumu Eguchi

**Affiliations:** ^1^ Kyushu Study Group of Liver Surgery Japan; ^2^ Department of Surgery and Science, Graduate School of Medical Sciences Kyushu University Fukuoka Japan; ^3^ Department of Surgery Saga University Faculty of Medicine Saga Japan; ^4^ Division of Hepatobiliary and Pancreatic Surgery, Department of Surgery Kurume University Kurume Japan; ^5^ Department of Gastroenterological, Breast, and Endocrine Surgery Yamaguchi University Graduate School of Medicine Ube Japan; ^6^ Department of Gastroenterological Surgery, Graduate School of Life Sciences Kumamoto University Kumamoto Japan; ^7^ Department of Surgery Saga‐Ken Medical Center Koseikan Saga Japan; ^8^ Department of Hepato‐Biliary‐Pancreatic Surgery, Clinical Research Institute National Hospital Organization Kyushu Medical Center Fukuoka Japan; ^9^ Division of Hepato‐Biliary‐Pancreas Surgery, Department of Surgery University of Miyazaki Faculty of Medicine Miyazaki Japan; ^10^ Department of Surgery National Hospital Organization Nagasaki Medical Center Nagasaki Japan; ^11^ Department of Gastroenterological and Pediatric Surgery Oita University Faculty of Medicine Graduate School of Medicine Oita Oita Japan; ^12^ Department of Surgery, Gastroenterology and Hepatology Center Kitakyushu City Yahata Hospital Kitakyushu Japan; ^13^ Department of Gastroenterological Surgery, Faculty of Medicine Fukuoka University Fukuoka Japan; ^14^ Department of Surgery Kagoshima Kouseiren Hospital Kagoshima Japan; ^15^ Department of Surgery Yamaga City Medical Center Yamaga Japan; ^16^ Department of Digestive and General Surgery, Graduate School of Medicine University of the Ryukyus Nishihara Japan; ^17^ Department of Surgery Nagasaki University Graduate School of Biomedical Sciences Nagasaki Japan

**Keywords:** direct‐acting antivirals, hepatitis C virus, hepatocellular carcinoma, interferon, sustained virological response

## Abstract

**Aim:**

We examine the impact of interferon (IFN)‐based and IFN‐free treatment on the prognosis of hepatocellular carcinoma (HCC) after hepatitis C virus (HCV) sustained virological response (SVR).

**Methods:**

Clinical information was collected on 311 cases of HCC after HCV‐SVR from 16 facilities affiliated with the Kyushu Liver Surgery Study Group. Clinical factors and the tumor microenvironment of HCC after SVR treatment with IFN‐based and IFN‐free treatments were analyzed.

**Results:**

No statistically significant differences were observed in the recurrence rate and overall survival (OS) between the two groups. Propensity score–matched analysis similarly showed no statistically significant differences in recurrence and OS. In the IFN‐based treatment group, OS time was significantly shorter for the programmed death‐ligand 1(PD‐L1)‐positive HCC than the PD‐L1‐negative HCC group (*p* = 0.0183). No significant difference was observed in the recurrence rate between PD‐L1‐positive and PD‐L1‐negative HCC groups. In the IFN‐based treatment group, the recurrence rate in the cluster of differentiation (CD) 8‐positive group was significantly lower than in the CD8‐negative group (*p* = 0.0292). There was no difference in OS time between the CD8‐positive and CD8‐negative groups. In the IFN‐free treatment group, PD‐L1 and CD8 were not associated with recurrence rate or OS.

**Conclusions:**

No statistically significant differences were observed in recurrence or OS rate after HCC resection in the IFN‐free treatment group compared with the IFN‐based treatment group. In the IFN‐based treatment group, PD‐L1 and CD8 expression on cancer cells might be prognostic factors.

**Abbreviations**
AFP: alpha‐fetoprotein
ALT: alanine transaminase
AST: aspartate transaminase
CD: cluster of differentiation
DAA: direct‐acting antivirals
DCP: *des*‐gamma‐carboxyprothrombin
DCV: daclatasvir
HCC: hepatocellular carcinoma
HCV: (hepatitis C virus)
IFN: interferon
IHC: immunohistochemical
IQR: interquartile range
OS: overall survival
PD‐L1: (programmed death‐ligand 1)
RBV: ribavirin
SOF: sofosbuvir
SVR: sustained virological response

## 1 Introduction

Hepatocellular carcinoma (HCC) is a prevalent malignancy on a global scale [1]. Cirrhosis induced by persistent hepatitis C virus (HCV) infection represents a major risk factor for HCC [2].

With the advent of direct‐acting antivirals (DAA) over the past decade, significant advancements have been achieved in the treatment of chronic hepatitis C. During the 2000s, pegylated interferon (IFN)‐based therapy yielded a virologic response rate of 40%–70%. In contrast, in the DAA era, the sustained virologic response (SVR) rate at 12 weeks has been reported to range from 90% to 98%, even among patients with advanced liver cirrhosis [3, 4, 5, 6]. Beyond viral eradication, IFN‐based therapy exerts immunomodulatory and anti‐proliferative effects, whereas DAA therapy achieves rapid viral clearance through direct inhibition of viral replication without known direct immune stimulation. Recent studies have reported elevated rates of HCC occurrence and recurrence following DAA therapy [7, 8, 9, 10]. However, there are some negative reports on the effectiveness of DAA treatment in the recurrence of HCC [11, 12], and the impact of antiviral treatment modality on tumor biology and postoperative outcomes remains controversial.

Therefore, in this multicenter retrospective study, we aimed to compare the clinicopathological characteristics, tumor microenvironment, and postoperative outcomes of HCC developing after SVR between patients treated with IFN‐based and IFN‐free regimens.

## 2 Methods

### 2.1 Patients

All 534 patients with HCC after SVR who underwent surgical resection between January 2000 and December 2019 at the 16 institutions belonging to the Kyushu Study Group of Liver Surgery (Kyushu University, Saga University, Kurume University, Kumamoto University, Yamaguchi University, Saga‐Ken Medical Center Koseikan, National Hospital Organization Kyushu Medical Center, National Hospital Organization Nagasaki Medical Center, Miyazaki University, Fukuoka University, Kitakyushu City Yahata Hospital, Yamaga City Medical Center, Kagoshima Kouseiren Hospital, Oita University, University of the Ryukyus, and Nagasaki University) were enrolled in this study. Although resection is typically recommended for early‐stage HCC, selected patients with Barcelona Clinic Liver Cancer (BCLC) stage B or limited stage C disease underwent upfront surgical resection based on preserved liver function and technical resectability at each participating center. No preoperative systemic therapy was administered in these cases. Patients diagnosed with HCC within 1 year after achieving SVR were excluded to minimize the inclusion of pre‐existing, radiologically undetectable tumors at the time of SVR confirmation and to better capture incident HCC developing after viral eradication. We collected tissues for immunological staining in 343 out of 534 cases. This study received approval from the Institutional Review Board of each participating institution. Postoperative monitoring comprised evaluations of alpha‐fetoprotein (AFP) and des‐gamma‐carboxyprothrombin (DCP) levels, along with ultrasonography, computed tomography, or magnetic resonance imaging at three‐month intervals.

### 2.2 Immunohistochemical (IHC) Staining

IHC staining for programmed death‐ligand 1 (PD‐L1) and Cluster of Differentiation 8 (CD8) was performed as previously described [13]. Stained slides were digitally scanned utilizing the NanoZoomer (Hamamatsu Photonics KK, Japan). PD‐L1 and CD8 staining were assessed by two independent, experienced researchers (K.T. and S.I.) who were blinded to the clinical status of the patients. The final evaluations were reached through consensus.

### 2.3 Statistical Analysis

Standard statistical methods were used to analyze descriptive statistics, including medians, frequencies, and percentages. The Mann–Whitney U test was utilized to compare continuous variables that did not follow a normal distribution, while categorical variables were assessed using the X^2^ test or Fisher's exact test. Cumulative overall survival (OS) and recurrence rates were calculated using the Kaplan–Meier method, and differences between the survival curves were evaluated using the log‐rank test. Recurrence rate was analyzed using standard time‐to‐event methods. Death was evaluated separately as an endpoint in overall survival and cancer‐specific survival analyses and was not modeled as a competing risk in the recurrence analysis. Time from SVR to HCC occurrence was evaluated in patients who developed HCC after achieving SVR and subsequently underwent surgical resection. Because the study population consisted exclusively of post‐SVR HCC cases, all included patients experienced the event of interest, and no censoring was applied. The distribution of time from SVR to HCC occurrence was illustrated using Kaplan–Meier curves, and differences between groups were assessed using the log‐rank test. Propensity score–matched analysis was performed because baseline patient characteristics differed between the IFN‐free and IFN‐based treatment groups. Propensity scores were estimated using a logistic regression model including baseline patient characteristics and tumor‐related variables. A 1:1 nearest‐neighbor matching without replacement was performed. Operative variables were not included to avoid over‐adjustment. Differences were deemed statistically significant at *p* < 0.05. All analyses were conducted using JMP software, version 15 (SAS Institute Inc., Cary, NC, USA).

## 3 Results

### 3.1 Patient Characteristics

**FIGURE 1:**
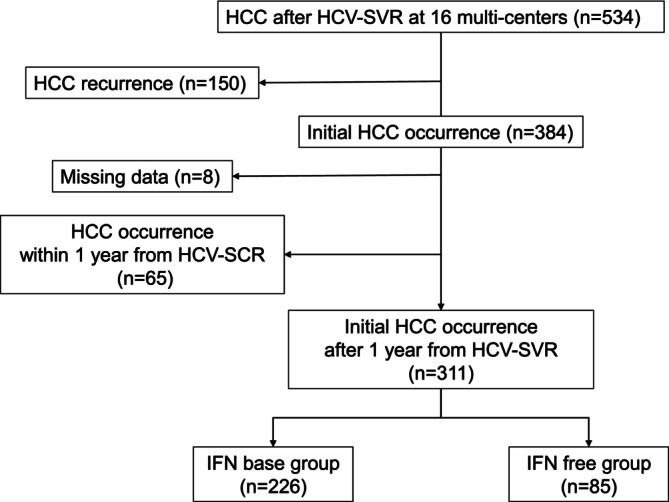
Flow diagram of the patient selection process. HCC, hepatocellular carcinoma; HCV, hepatitis C virus; IFN, interferon; SVR, sustained virological response.

The patient flow in this study is shown in Figure 1. From the 534 cases, we enrolled 311 cases in this study, excluding recurrent hepatocellular carcinoma cases (*n* = 150), missing data (*n* = 8), and HCC occurrence 1 year after HCV‐SVR (*n* = 65). The study population constituted 212 men and 99 women, with a median age of 71 years. The median body mass index before surgery was 22.8 kg/m^2^ (interquartile range (IQR): 21.4–25.7). Of the 311 patients, 5 (1.6%) were seropositive for hepatitis B surface antigen. The largest tumors had a median diameter of 2.6 cm (IQR: 1.7–4.0 cm). In this cohort, 52 (16.7%) had multiple tumors; 38 (12.2%) had BCLC stage B or C disease; 77 (24.8%) showed poor pathological differentiation; 91 (29.2%) had microscopic diagnoses of vascular invasion; 19 (6.1%) had microscopic intrahepatic metastases; and 167 (53.7%) had stage 3–4 pathological liver fibrosis. The median AFP and DCP levels were 6.2 (IQR: 3.1–86.7) ng/mL and 59.5 (IQR: 23–468) U/mL, respectively.

### 3.2 Antiviral Treatment

Of the 311 patients, 226 were treated by an IFN‐based treatment, and 85 were treated by an IFN‐free treatment. The IFN‐free tretment included asunaprevir plus daclatasvir (DCV) (*n* = 47), sofosbuvir (SOF) plus ribavirin (RBV) (*n* = 5), paritaprevir/ombitasvir/ritonavir (*n* = 2), SOF/ledipasvir (*n* = 29) and glecaprevir/pibrentasvir (*n* = 2) (Table 1).

**TABLE 1:** Interferon‐Free treatment.

MEdicine	IFN free treatment (*n* = 85) (%)
asunaprevir + daclatasvir	47 (55.3)
sofosbuvir + ribavirin	5 (5.9)
Paritaprevir + ombitasvir + ritonavir	2 (2.4)
Sofosbuvir + ledipasvir	29 (34.3)
Glecaprevir + pibrentasvir	40 (47.1)

### 3.3 Relationships Between Clinicopathological Factors and Treatment

The baseline characteristics and laboratory data of the IFN‐based treatment or IFN‐free treatment are shown in Table 2. The IFN‐free treatment group consisted of more women (*p* < 0.0001), lower platelet count (*p* < 0.0001), lower aspartate transaminase (AST) level (*p* = 0.0335), lower alanine transaminase (ALT) level (*p* < 0.0001), lower γ‐glutamyltransferase level (*p* = 0.0011), higher Fib‐4 index (*p* < 0.0001), higher AST to ALT ratio (*p* = 0.0026), and higher AST to platelet ratio index (*p* = 0.0195).

**TABLE 2:** Background characteristics of patients who underwent liver resection.

Variable	IFN base (*n* = 226)	IFN free (*n* = 85)	*p*
Age (years)	71 (64–75)	72 (67–79)	0.0187
Sex, male/female	169/57	43/42	< 0.0001
BMI (kg/m^2^)	23.5 (21.6–25.7)	22.8 (20.7–25.2)	0.0680
Diabetes mellitus (%)	61 (26.9%)	24 (28.2%)	0.8263
Alcohol (%)	40 (17.9%)	14 (16.4%)	0.7622
Varices (%)	24 (10.8%)	12 (14.1%)	0.4282
HBs‐Ag positive (%)	5 (2.2%)	0 (0%)	0.1668
Albumin (g/dL)	4.2 (3.9–4.5)	4.2 (4.0–4.5)	0.8906
Total bilirubin (mg/dL)	0.8 (0.6–1.0)	0.8 (0.6–1.0)	0.9620
Platelet (x10^4^/μL)	17.7 (14.3–22.0)	13.7 (10.3–17.8)	< 0.0001
AST (IU/L)	27 (22–33)	25 (20–30)	0.0335
ALT (IU/L)	21 (15–33)	16 (13–22)	< 0.0001
γGTP	36 (25–63)	27 (18–44)	0.0011
ALBI score	–2.84 (–2.59 to –3.11)	–2.78 (–2.69 to –3.03)	0.8055
ALBI grade, 1/2/3	170/56/0	71/14/0	0.1179
Modified ALBI grade, 1/2a/2b/3	170/37/19/0	71/10/4/0	0.2778
FIB4 index	2.32 (1.77–2.94)	3.21 (2.22–4.62)	< 0.0001
AAR	1.27 (0.97–1.52)	1.42 (1.14–1.79)	0.0026
APRI	0.50 (0.39–0.69)	0.62 (0.40–0.91)	0.0195

*Note:* Data are presented as *n* (%) or median (interquartile range).

Abbreviations: AAR, AST to ALT ratio; ALBI, albumin–bilirubin; ALT, alanine transaminase; APRI, AST to platelet ratio index; AST, aspartate transaminase; BMI, body mass index; GTP, glutamyltransferase; HBs‐Ag, hepatitis B surface antigen; IFN, interferon.

We showed the tumor, perioperative, and pathological characteristics of the IFN‐based treatment and IFN‐free treatment in Table 3. The IFN‐free treatment group correlated with small tumor size (*p* < 0.0001), a low rate of imaging vascular invasion (*p* = 0.0114), early Barcelona Clinic Liver Cancer stage (*p* = 0.0131), within Milan criteria (*p* = 0.0008), a high rate of laparoscopic surgery (*p* < 0.0001), a low rate of anatomical liver resection (*p* = 0.0117), shorter duration of surgery (*p* = 0.0316), few of blood loss (*p* = 0.0154), shorter of postoperative hospital stay (*p* = 0.0005), a high rate of single nodular type (*p* = 0.0102), and microscopic liver fibrosis (*p* < 0.0001).

**TABLE 3:** Tumor, perioperative, and pathological characteristics of patients who underwent liver resection.

Variable	IFN base (*n* = 226)	IFN free (*n* = 85)	*p*
AFP (ng/mL)	5.9 (3.0–75.1)	7.3 (3.1–125)	0.4692
DCP (mAU/mL)	61 (23–776)	49 (21–174)	0.1155
Tumor size (cm)	2.9 (1.8–4.5)	2.1 (1.5–3.0)	< 0.0001
Solitary/Multiple	188/38	71/14	0.9423
Imaging vascular invasion (%)	21 (9.2%)	1 (1.1%)	0.0114
Lymph node metastasis (%)	1 (0.4%)	0 (0%)	0.5390
Extrahepatic metastasis (%)	1 (0.4%)	0 (0%)	0.5390
BCLC staging, B or C	34 (15.0%)	4 (4.7%)	0.0131
Within Milan criteria (%)	168 (74.3%)	78 (91.7%)	0.0008
Laparoscopic surgery (%)	83 (36.7%)	56 (65.8%)	< 0.0001
Anatomical liver resection (%)	152 (67.2%)	44 (51.7%)	0.0117
Duration of surgery (min)	320 (228–420)	281 (217–357)	0.0316
Blood loss (g)	343 (100–648)	200 (55–447)	0.0154
Intraoperative blood transfusion (%)	21 (9.2%)	8 (9.4%)	0.9742
Postoperative complication (%)	31 (13.7%)	7 (8.2%)	0.1884
Postoperative hospital stay (days)	14 (10–18)	11 (9–15)	0.0005
Gross classification, single nodular type (%)	126 (55.7%)	61 (71.7%)	0.0102
Poor differentiation (%)	60 (26.5%)	17 (20.0%)	0.2331
Microscopic vascular invasion (%)	71 (31.4%)	20 (23.5%)	0.1731
Microscopic intrahepatic metastasis (%)	14 (6.1%)	5 (5.8%)	0.9184
F3 or F4 (%)	88 (38.9%)	56 (65.8%)	< 0.0001

*Note:* Data are presented as *n* (%) or median (interquartile range).

Abbreviations: AFP, alpha‐fetoprotein; BCLC, Barcelona Clinic Liver Cancer; DCP, des‐gamma‐carboxyprothrombin; F, fibrosis; IFN, interferon.

### 3.4 Analyses of Prognosis in HCC After SVR by IFN‐Based Treatment or IFN‐Free Treatment

**FIGURE 2:**
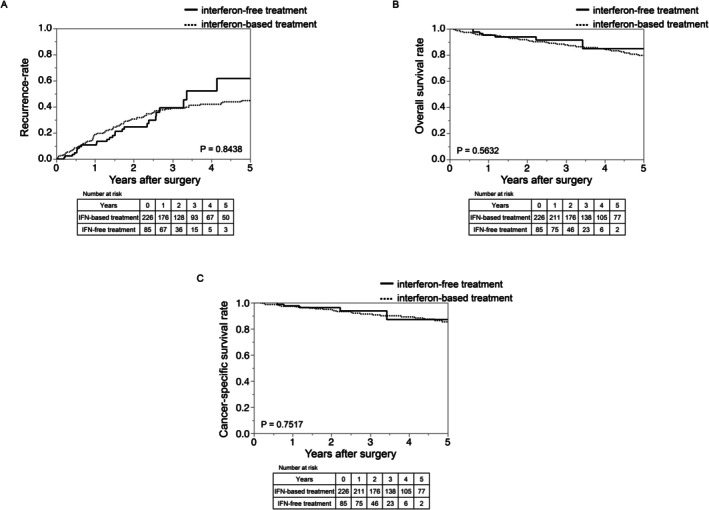
Recurrence rate (A), overall survival rate (B), and cancer‐specific survival rate (C) of patients with hepatocellular carcinoma in the interferon‐free or interferon‐based treatment groups. Data were analyzed using Kaplan–Meier analysis, and statistical significance was determined by a log‐rank test. IFN, interferon.

**FIGURE 3:**
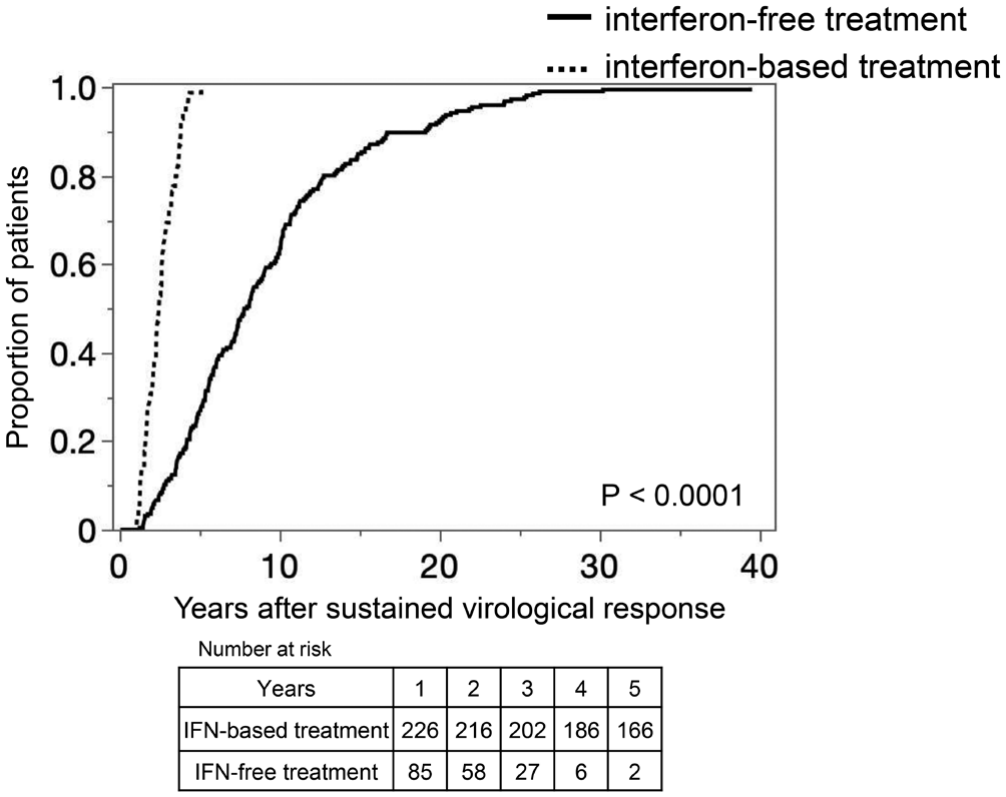
Distribution of time from sustained virological response (SVR) to hepatocellular carcinoma (HCC) occurrence in surgically treated post‐SVR HCC cases. Because this cohort includes only patients who developed HCC after SVR, the curve represents the distribution of time to HCC occurrence rather than incidence. Data were analyzed using the Kaplan–Meier method, and statistical significance was assessed using the log‐rank test. IFN, interferon.

**FIGURE 4:**
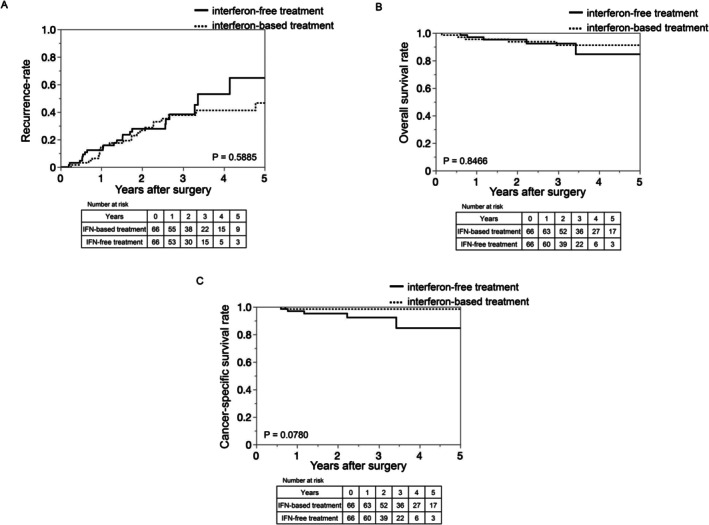
Recurrence rate (A), overall survival rate (B), and cancer‐specific survival rate (C) of patients with hepatocellular carcinoma in the interferon‐free or interferon‐based treatment groups after propensity score matching. Data were analyzed using Kaplan–Meier analysis, and statistical significance was determined by a log‐rank test. IFN, interferon.

We next assessed the associations between the treatment of HCV and the prognosis of patients with HCC after hepatic resection using Kaplan–Meier curves. The results demonstrated that the recurrence rate, OS rate, and cancer‐specific survival rate were not significantly different between the IFN‐based group and the IFN‐free group (Figure 2A–C). Among patients who developed HCC after achieving SVR and underwent surgical resection, the time from SVR to HCC occurrence differed between treatment groups. Kaplan–Meier curves demonstrated a significantly shorter interval from SVR to HCC occurrence in the IFN‐free group compared with the IFN‐based therapy group (Figure 3) (*p* < 0.0001). Propensity scores were calculated with age, sex, platelet, AST, ALT, γGTP, tumor size, imaging vascular invasion, BCLC B or C, and within Milan criteria, and propensity score matching (1:1) was performed (Table 4 and Table 5). Sixty‐six patients were retained in the matched cohort, and no statistically significant differences were observed at the time in a comparison between the 2 groups of the recurrence rate, OS rate, and cancer‐specific survival rate (Figure 4A–C).

**TABLE 4:** Background characteristics of patients who underwent liver resection after propensity score matching.

Variable	IFN base (*n* = 66)	IFN free (*n* = 66)	*p*
Age (years)	72 (69–76)	71 (67–77)	0.5427
Sex, male/female	35/31	40/26	0.3796
BMI (kg/m^2^)	23.0 (21.4–25.4)	22.7 (20.7–25.8)	0.7107
Diabetes mellitus (%)	21 (31.8%)	22 (33.3%)	0.8527
Alcohol (%)	6 (9.1%)	13 (19.7%)	0.0826
Varices (%)	9 (13.6%)	9 (13.6%)	1.0000
HBs‐Ag positive (%)	0 (0%)	0 (0%)	1.0000
Albumin (g/dL)	4.3 (4.0–4.5)	4.2 (4.0–4.5)	0.6415
Total bilirubin (mg/dL)	0.8 (0.7–1.1)	0.8 (0.6–1.0)	0.2211
Platelet (x10^4^/μL)	16.0 (13.3–18.7)	15.2 (11.6–19.1)	0.3711
AST (IU/L)	24 (20–28)	24 (20–31)	0.9255
ALT (IU/L)	19 (13–25)	16 (14–24)	0.3149
γGTP	34 (24–47)	33 (20–49)	0.5786
ALBI score	–2.87 (–2.63 to –3.03)	–2.77 (–2.70 to –3.04)	0.7953
ALBI grade, 1/2/3	52/14/0	58/8/0	0.1611
Modified ALBI grade, 1/2a/2b/3	52/10/4/0	58/5/3/0	0.3436
FIB4 index	2.56 (1.94–3.50)	2.78 (1.97–4.00)	0.3102
AAR	1.31 (1.09–1.55)	1.36 (1.12–1.69)	0.3699
APRI	0.49 (0.40–0.66)	0.55 (0.35–0.90)	0.3785

*Note:* Data are presented as *n* (%) or median (interquartile range).

Abbreviations: AAR, AST to ALT ratio; ALBI, albumin–bilirubin; ALT, alanine transaminase; APRI, AST to platelet ratio index; AST, aspartate transaminase; BMI, body mass index; GTP, glutamyltransferase; HBs‐Ag, hepatitis B surface antigen; IFN, interferon.

**TABLE 5:** Tumor, perioperative, and pathological characteristics of patients who underwent liver resection after propensity score matching.

Variable	IFN base (*n* = 66)	IFN free (*n* = 66)	*p*
AFP (ng/mL)	5.4 (3.0–50)	7.9 (3.5–153)	0.2889
DCP (mAU/mL)	28 (18–123)	43 (22–152)	0.2074
Tumor size (cm)	2.2 (1.5–3.2)	2.3 (1.7–3.0)	0.5339
Solitary/Multiple	53/13	54/12	0.8242
Imaging vascular invasion (%)	1 (1.5%)	1 (1.5%)	1.0000
Lymph node metastasis (%)	0 (0%)	0 (0%)	1.0000
Extrahepatic metastasis (%)	0 (0%)	0 (0%)	1.0000
BCLC staging, B or C	3 (4.5%)	3 (4.5%)	1.0000
Within Milan criteria (%)	58 (87.8%)	60 (90.9%)	0.5718
Laparoscopic surgery (%)	41 (62.1%)	39 (59.0%)	0.7216
Anatomical liver resection (%)	39 (59.0%)	36 (54.5%)	0.5981
Duration of surgery (min)	283 (211–382)	283 (200–362)	0.9891
Blood loss (g)	170 (32–428)	182 (55–504)	0.2697
Intraoperative blood transfusion (%)	4 (6.0%)	6 (9.0%)	0.5106
Postoperative complication (%)	6 (9.0%)	6 (9.0%)	1.0000
Postoperative hospital stay (days)	11 (8–15)	11 (9–15)	0.8371
Gross classification, single nodular type (%)	39 (59.0%)	45 (68.1%)	0.2776
Poor differentiation (%)	14 (21.2%)	14 (21.2%)	1.0000
Microscopic vascular invasion (%)	12 (18.1%)	16 (24.2%)	0.3944
Microscopic intrahepatic metastasis (%)	3 (4.5%)	5 (7.8%)	0.4657
F3 or F4 (%)	35 (53.0%)	42 (63.6%)	0.2165

*Note:* Data are presented as *n* (%) or median (interquartile range).

Abbreviations: AFP, alpha‐fetoprotein; BCLC, Barcelona Clinic Liver Cancer; DCP, des‐gamma‐carboxyprothrombin; F, fibrosis; IFN, interferon.

### 3.5 The Survival Curves

**FIGURE 5:**
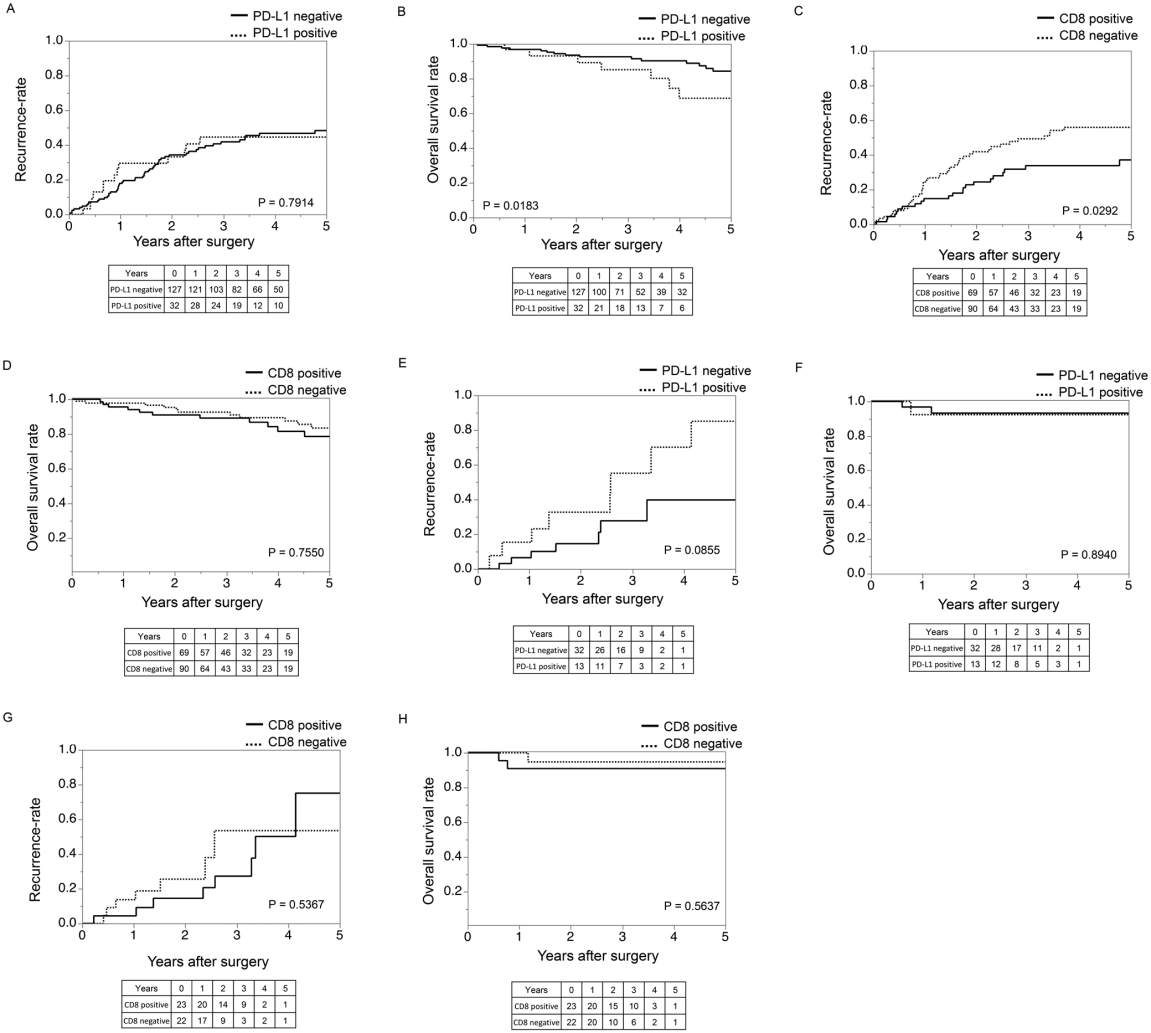
Recurrence rate (A) and overall survival rate (B) of patients receiving interferon‐based treatment were analyzed based on programmed death‐ligand 1 (PD‐L1)‐positive and ‐negative staining of tissue samples. Recurrence rate (C) and overall survival rate (D) of patients receiving interferon‐free treatment were analyzed based on cluster of differentiation 8 (CD8)‐positive and ‐negative staining of tissue samples. Recurrence rate (E) and overall survival rate (F) of patients receiving interferon‐free treatment were analyzed based on PD‐L1‐positive and ‐negative staining of tissue samples. Recurrence rate (G) and overall survival rate (H) of patients in interferon‐free treatment were analyzed based on CD8‐positive and ‐negative staining of tissue samples. Data were analyzed using Kaplan–Meier analysis, and statistical significance was determined by a log‐rank test. CD8, cluster of differentiation 8; PD‐L1, programmed death‐ligand 1.

Next, we performed immunohistochemical staining for PD‐L1 and CD8 in 204 of 311 HCC tissues (Figure S1). The cutoff was 1% for PD‐L1 and 13 counts for CD8, as previously reported [13]. Among the 204 samples, 45 (22.1%) were positive for PD‐L1 and 92 (45.1%) were positive for CD8. In the IFN‐based treatment group, the Kaplan–Meier curves demonstrate that OS time was significantly shorter for the PD‐L1‐positive HCC, compared with the PD‐L1‐negative HCC (*p* = 0.0183). No statistically significant differences were observed in recurrence rate between PD‐L1‐positive and PD‐L1‐negative HCC (Figure 5A,B). In the IFN‐based treatment group, the recurrence rate in the CD8‐positive group was significantly lower than in the CD8‐negative group (*p* = 0.0292). There was no significant difference in OS time between the CD8‐positive and CD8‐negative groups (Figure 5C,D). In the IFN free treatment group, PD‐L1 and CD8 were not associated with recurrence rate or OS (Figure 5E–H).

## 4 Discussion

The results of the present study using a multicenter study cohort revealed that the patients in whom HCV infection was successfully eradicated by IFN‐free treatment after curative treatment of initial HCC had a similar recurrence rate, OS, and cancer‐specific survival rate as those who underwent IFN‐based treatment.

Recent studies have reported a high rate of tumor recurrence following successful DAA therapy for chronic hepatitis C in patients with a prior history of treated HCC [14]. Nagata et al. demonstrated that the risks of early HCC occurrence and recurrence after viral eradication were similar between IFN‐based and IFN‐free treatments [15]. One meta‐analysis found no significant differences in HCC occurrence or recurrence rates between patients treated with IFN‐based and IFN‐free treatment [16]. In the present study, we showed that there were no significant differences in recurrence rate, OS rate, and cancer‐specific survival rate between the IFN‐based treatment group and the IFN‐free treatment group in both the all‐cohort and the propensity score‐matched cohort.

In the present study, the platelet count was lower, and the FIB4 index was higher in the IFN‐free treatment group compared to the IFN‐based treatment group. IFN‐free therapy has the potential to rapidly alleviate liver inflammation and fibrosis in HCV patients following SVR [17]. IFN‐free therapy appears to exert a time‐dependent influence, initially leading to viral clearance and resolution of liver inflammation, followed by more gradual modifications in structural hepatic lesions. These enhancements contributed to improved liver function, subsequently leading to the alleviation of cognitive dysfunction and portal hypertension [18]. The difference observed in the FIB‐4 index and platelet count between IFN‐free treatment and IFN‐based treatment was thought to be due to differences in patient background before treatment. In Japan, during the IFN era, treatment selection was influenced not only by drug availability but also by patient factors such as liver function and platelet count. IFN‐based therapy was often not suitable for patients with advanced liver disease or thrombocytopenia, and such patients were more likely to receive IFN‐free therapy when it became available. We hypothesized that this was because IFN treatment was not appropriate for patients with low platelet counts, and IFN‐free treatment was appropriate for patients with compensated cirrhosis.

Patients in the IFN‐free group presented after 3.35 years on average, while patients in the IFN‐based group presented after about seven years [19]. In the present study, we observed a difference in the distribution of time from SVR to HCC occurrence between treatment groups within a surgically treated post‐SVR HCC cohort. One possible explanation for this finding was the difference in background liver condition between treatment eras, as patients in the IFN‐free group tended to have more advanced liver disease at the time of antiviral therapy. However, this interpretation should be made with caution because the present analysis was restricted to patients who developed HCC after SVR and underwent surgical resection.

We showed that in the IFN‐based treatment group, PD‐L1 expression on cancer cells was associated with OS, and CD8 was associated with recurrence rate. Furthermore, in the IFN‐free treatment group, PD‐L1 and CD8 in cancer cells were not associated with either recurrence rate or OS. IFN‐based therapy has been reported to partially restore the impaired immune response, leading to a reduction in both circulating and liver‐infiltrating regulatory T‐cells following treatment [20]. In this regard, the delayed clearance of the virus, coupled with the immunomodulatory and anti‐proliferative effects of IFN, might play a role in facilitating a more gradual recovery of immune function following SVR. Conversely, it has been hypothesized that the rapid elimination of HCV through IFN‐free therapy may disrupt immune reconstitution within the liver microenvironment. This disruption, driven by the abrupt decline in viral load, could potentially compromise immune surveillance, thereby influencing the occurrence or recurrence of HCC [21]. Therefore, it was possible that the cancer growth and carcinogenesis patterns were different between the IFN‐based treatment group and the IFN‐free treatment group. The immune phenotyping in the present study was limited to PD‐L1 expression and CD8‐positive lymphocyte infiltration. Although these markers were clinically important, a more comprehensive evaluation, including additional tumor and immune markers, would provide a deeper understanding of tumor biology and would warrant further investigation.

There were some limitations to our study. The observation period was different between the IFN‐free treatment group and the IFN‐based treatment group. In addition, the present analysis was limited to surgically treated HCC cases developing after SVR and does not reflect population‐based HCC incidence after antiviral therapy. Furthermore, if the observation period was long, there was a possibility that HCC might develop from lifestyle‐related diseases. It is necessary to continue to follow up and investigate patients in the IFN‐free treatment group. Recurrence was analyzed without explicitly modeling death as a competing risk. Although elderly patients with cirrhosis and HCC are at increased risk of mortality, OS and cancer‐specific survival were comparable between treatment groups in this study, suggesting that differential mortality is unlikely to have markedly influenced the comparison of recurrence outcomes. Nevertheless, competing risk analyses may be warranted in future studies with longer follow‐up. In Japan, sorafenib was introduced in 2009, and immune checkpoint inhibitor–based therapy became available in 2020. Because the present cohort spans these treatment eras, post‐recurrence management may have differed over time. This temporal heterogeneity represents a limitation of the study.

In conclusion, no statistically significant differences were observed in recurrence rate, OS rate, and cancer‐specific survival rate after HCC resection in the IFN‐free treatment group compared with the IFN‐based treatment group. These findings suggest that postoperative outcomes of surgically treated HCC developing after SVR are comparable between treatment eras. Follow‐up after HCC resection was necessary for both treatment groups.

## Author Contributions

Shinji Itoh conceived and designed the study. Shinji Itoh, Norifumi Iseda, Tomoharu Yoshizumi, Takao Ide, Hirokazu Noshiro, Hisamune Sakai, Toru Hisaka, Masao Nakashima, Hiroaki Nagano, Hirohisa Okabe, Hiromitsu Hayashi, Atsushi Miyoshi, Kenji Kitahara, Yuko Takami, Atsushi Nanashima, Tamotsu Kuroki, Yuichi Endo, Kohji Okamoto, Masatoshi Kajiwara, Masahiko Sakoda, Toru Beppu, Mitsuhisa Takatsuki, and Susumu Eguchi collected and analyzed the data. Shinji Itoh and Norifumi Iseda drafted the manuscript. All authors contributed to data interpretation, critically revised the manuscript, and approved the final version of the manuscript.

## Ethics Statement

This study was approved by the Ethics Committee of Kyushu University (approval code: 2020–345), and it conforms to the provisions of the Declaration of Helsinki.

## Consent

We obtained informed consent from all patients.

## Conflicts of Interest

The authors declare no conflicts of interest.

## Supporting information


**Figure S1:** Immunohistochemical staining of PD‐L1 and CD8 expression in hepatocellular carcinoma tissue. (A) Negative and positive membrane staining of PD‐L1. (B) Low and high CD8 expression in tumor‐infiltrating T cells. PD‐L1; programmed death‐ligand 1, CD8; Cluster of Differentiation 8.

## Data Availability

No data are available.
