# Supplementary material for: Comparison of Interferon‐Based and Interferon‐Free Treatments on the Prognosis of Hepatocellular Carcinoma After Hepatitis C Virus‐Sustained Virological Response: A Multicenter Study
Source: Cancer Med. 2026 Jun 11;15(6):e71963. doi: 10.1002/cam4.71963 (PMC13254683; doi:10.1002/cam4.71963)
Supplement: Supplementary file 1 — Figure S1: Immunohistochemical staining of PD‐L1 and CD8 expression in hepatocellular carcinoma tissue. (A) Negative and positive membrane staining of PD‐L1. (B) Low and high CD8 expression in tumor‐infiltrating T cells. PD‐L1; programmed death‐ligand 1, CD8; Cluster of Differentiation 8. [file CAM4-15-e71963-s001.pdf]

Supplementary figure

A

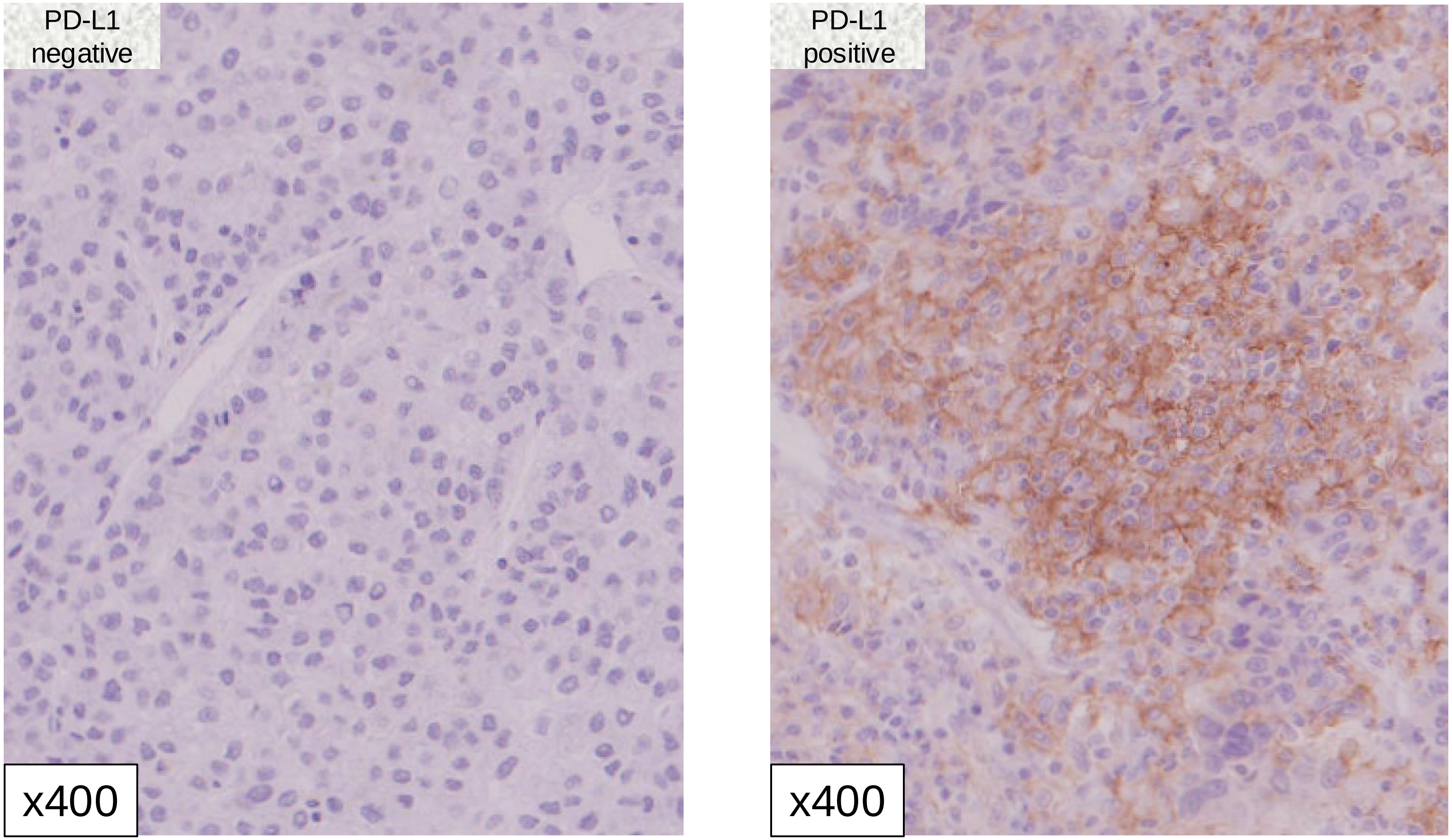

B

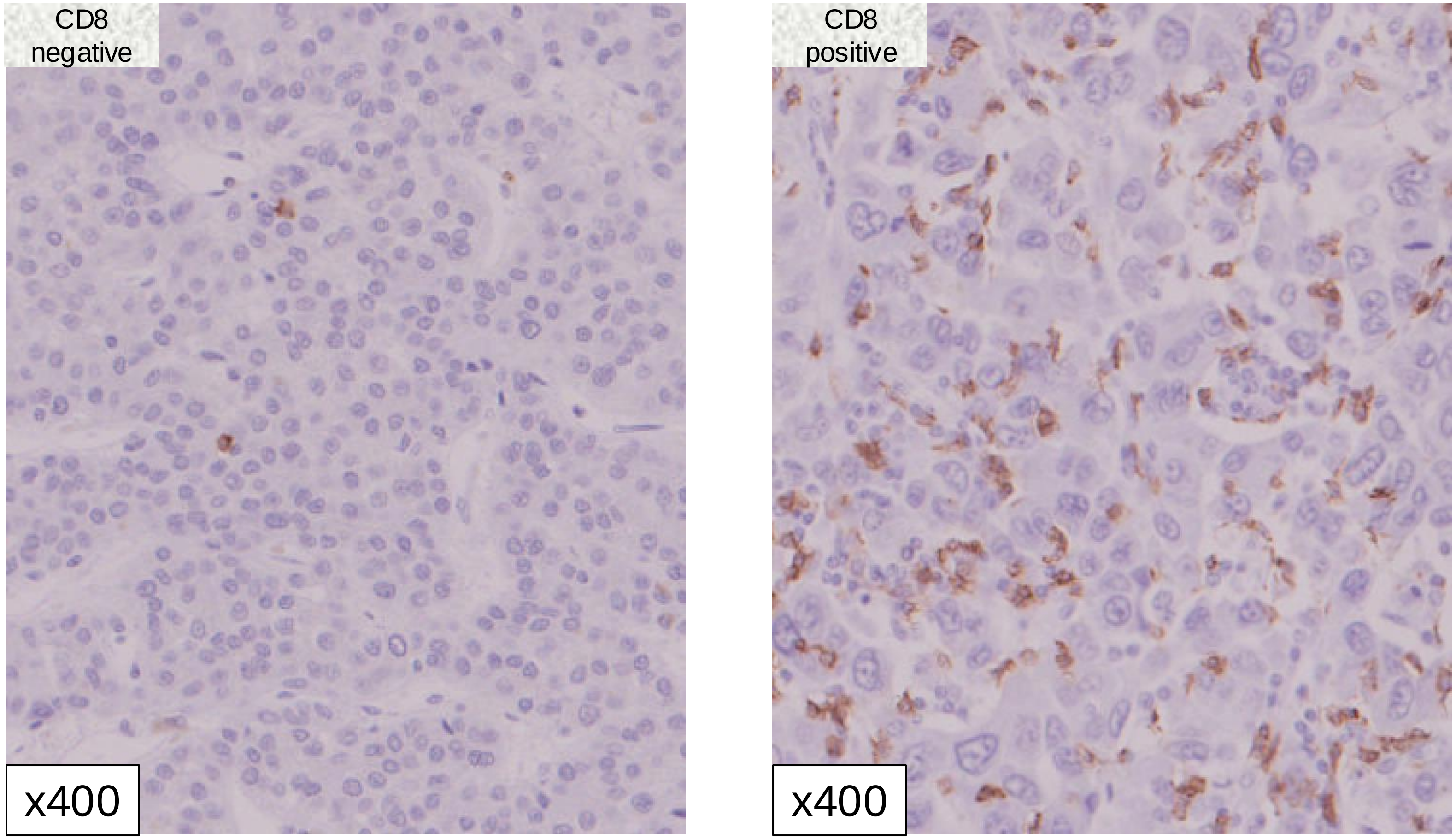

Supplementary figure. Immunohistochemical staining of PD-L1 and CD8 expression in hepatocellular carcinoma tissue. (A) Negative and positive membrane staining of PD-L1. (B) Low and high CD8 expression in tumor-infiltrating T cells. PD-L1; programmed death-ligand 1, CD8; Cluster of Differentiation 8.
